# Can Surgical Approach and Postoperative Factors Impact Survival in Rectal Cancer? Robotic Versus Laparoscopic Insights

**DOI:** 10.1002/cam4.71453

**Published:** 2025-12-17

**Authors:** Ahmed Abdelsamad, Seyidali Mirzazada, Karsten Ridwelski, Mohamad Nour Nasif, Florian Gebauer

**Affiliations:** ^1^ Department of Surgery II University of Witten/Herdecke Witten Germany; ^2^ Oncological Surgery Department Knappschaft Vest‐Hospital Recklinghausen Germany; ^3^ General, Vascular‐ and Gastrointestinal Surgery Department Magdeburg Hospital Magdeburg Germany; ^4^ Faculty of Medicine Aleppo University Aleppo Syria; ^5^ Oncological Surgery Department Helios University Hospital Wuppertal Germany

**Keywords:** 10‐year‐long‐term outcomes, complications, conversion, laparoscopic surgery, perineural invasion, rectal cancer, robotic surgery, survival

## Abstract

**Background:**

This study aimed to evaluate the long‐term oncologic outcomes of robotic‐assisted versus laparoscopic surgery in non‐metastatic patients with locally advanced rectal cancer and to identify prognostic factors influencing overall survival (OS) and disease‐free survival (DFS).

**Material and Methods:**

In this retrospective cohort study, 74 patients with mid or low rectal cancer underwent either laparoscopic (Gr. 1) (*n* = 28) or robotic‐assisted (Gr. 2) (*n* = 46) surgery over 10 years. Baseline characteristics, surgical details, postoperative complications, and survival outcomes were analyzed. Multivariate Cox regression was used to identify independent predictors of OS and DFS.

**Results:**

Both groups had no significant difference in hospital stay, conversion rates, or postoperative complications. Multivariate analysis revealed that robotic surgery was independently associated with improved OS (HR: 2.651; *p* = 0.019). Other significant predictors of poor OS included tumor grade G3, perineural invasion, and postoperative complications. For DFS, perineural invasion, postoperative complications, conversion to open surgery, and tumor recurrence were associated with worse outcomes. Restoration of bowel continuity via end‐to‐end anastomosis was linked to improved survival.

**Conclusions:**

Robotic‐assisted surgery offers comparable, and in some aspects superior, long‐term oncologic outcomes to laparoscopic surgery for locally advanced rectal cancer. Independent predictors of poor survival included high‐grade tumors, perineural invasion, conversion to open surgery, and postoperative complications. Surgical technique, selection criteria, and perioperative care remain crucial for optimizing outcomes.

**Abbreviations**
3D: tri‐dimensional
AJCC: American Joint Committee on Cancer
APR: abdominoperineal resection
ASA: American Society of Anesthesiologists
CRM: circumferential margin
DFS: disease free survival
EEA: end‐to‐end anastomosis
eTME: extended mesorectal excision
G: grade
Gr.: group
LAR: low anterior resection
LARC: locally advanced rectal cancer
LLND: lateral lymph node dissection
LLNs: lateral lymph nodes
LVI: lymphovascular invasion
OS: overall survival
Pn: perineural
R: resection margin
SOP: standard operating procedures
TME: total mesorectal excision
Vs.: versus

## 1 Introduction

The surgical management of locally advanced rectal cancer, particularly tumors located in the middle and lower third of the rectum, presents unique technical and oncological challenges due to the confined space of the pelvis, the proximity to critical neurovascular structures, and the importance of achieving clear margins while preserving function [1].

In rectal cancer surgery, it is not merely a question of what is removed, but rather what is left behind—underscoring the vital balance between oncologic radicality and the preservation of surrounding structures critical to postoperative function and long‐term outcomes [2].

While total mesorectal excision (TME) remains the cornerstone of curative rectal cancer surgery, the role of lateral lymph node dissection (LLND) continues to be debated. In Western countries, lateral pelvic lymph nodes (LLNs) are often considered sites of distant metastases and typically managed with neoadjuvant chemoradiotherapy (nCRT) or total neoadjuvant therapy (TNT). In contrast, Japanese protocols advocate for routine LLND in patients with lower rectal tumors, especially those below the peritoneal reflection, as studies have shown improved local control in selected cases [3]. For example, the JCOG0212 trial demonstrated a significantly lower local recurrence rate in patients who underwent LLND in addition to TME, although no significant difference in overall survival (OS) was observed [4]. Moreover, recent studies have suggested that LLND may benefit patients with clinically suspicious lateral nodes, particularly when MRI indicates a short‐axis diameter ≥ 7 mm [5, 6]. These differences underscore the necessity to evaluate the indications and outcomes of LLND in the context of both regional practice patterns and evolving evidence.

In contrast, a recent meta‐analysis in patients with locally advanced rectal cancer and clinically enlarged lateral lymph nodes after neoadjuvant chemoradiation showed no significant oncologic advantage of extended mesorectal excision over standard TME. The findings advocate for a tailored surgical approach based on tumor biology and response to treatment. Notably, international guidelines remain inconsistent—Japanese protocols support prophylactic dissection, German guidelines oppose it, and the AJCC staging system offers limited clarity—underscoring the need for global consensus [7].

In recent decades, minimally *invasive surgical techniques*, particularly laparoscopic TME, have been widely adopted and shown to offer benefits in terms of reduced perioperative morbidity, shorter hospital stays, and faster recovery, while maintaining oncologic safety [8, 9, 10]. However, laparoscopic rectal surgery is associated with limitations in instrument articulation and visualization, especially in narrow male pelvises or obese patients [8, 11]. To overcome these limitations, *robotic‐assisted surgery* has emerged as a promising alternative, offering improved ergonomics, enhanced three‐dimensional visualization, and greater dexterity, potentially translating into more precise dissection, better preservation of autonomic nerves, and improved oncologic outcomes [12].

Several studies have reported favorable short‐term outcomes with robotic rectal surgery, including lower conversion rates and improved functional results [12, 13, 14, 15, 16]. However, its long‐term oncologic efficacy remains less well defined and continues to be a subject of investigation [17].

Transanal total mesorectal excision (Ta‐TME) has emerged as an innovative minimally invasive approach for mid‐to‐low rectal cancers, aiming to overcome the technical limitations of conventional TME, particularly in anatomically challenging cases such as obese patients or those with a narrow pelvis. However, it is most beneficial in early‐stage tumors located at least 4 cm from the anal verge, without bulky tumors or advanced local disease. In these selected patients, Ta‐TME has been associated with favorable oncologic and functional outcomes, comparable to other techniques. Given its rising role in rectal cancer surgery, it is important to position Ta‐TME alongside other minimally invasive techniques when evaluating modern surgical approaches [18].

In addition to the surgical approach, several *pathologic and perioperative factors* have been identified as critical prognostic indicators in rectal cancer [19]. Yet, the interaction between these variables and the surgical modality in determining long‐term survival outcomes has not been clearly established.

Therefore, the present study was conducted to *compare long‐term oncologic outcomes*—specifically OS and disease‐free survival (DFS)—between robotic‐assisted and laparoscopic surgery in patients with *locally advanced rectal cancer*. Furthermore, this study aimed to identify *independent prognostic factors* that influence survival outcomes. Understanding these associations may help refine patient selection and optimize treatment strategies in the era of precision rectal cancer surgery.

## 2 Materials and Methods

Between 2014 and 2018, a retrospective, comparative cohort study was conducted at our Institution. The study population included patients with locally advanced adenocarcinoma of the middle or lower third of the rectum, all of whom underwent curative‐intent minimally invasive surgery. Patients were stratified into two groups according to the surgical approach—TME: the laparoscopic group (Group 1) and the robotic‐assisted group (Group 2), operated on using the Da Vinci Si HD Robotic System (Intuitive Surgical Inc., Sunnyvale, CA).

To ensure a consistent and homogenous dataset, we restricted the study period to 2014–2018. This timeframe precedes the adoption of a new robotic platform (Da Vinci Xi) and the establishment of a new surgical team. It also coincides with complete data availability prior to the transition to a new documentation system, which limited access to later records.

All procedures were performed by the same experienced surgical team using a standardized operative technique and perioperative management protocol, following institutional standard operating procedures (SOP).

Inclusion criteria for the study were: histologically confirmed, non‐metastatic, locally advanced rectal cancer (cT3–T4 and/or N+), located in the middle or lower third of the rectum (≤ 12 cm from the anal verge); adequate preoperative sphincter function and continence; and suitability for elective major surgery classified as ASA I–III.

Exclusion criteria comprised tumor location < 2 cm from the anal verge, ASA IV classification, emergency presentations such as obstruction or perforation, histologically undifferentiated tumors, presence of distant metastases at the time of diagnosis, and patient refusal to participate in long‐term follow‐up.

All patients underwent standardized preoperative staging including pelvic MRI and thoracoabdominal CT and rigid rectoscopy. Neoadjuvant chemoradiotherapy (nCRT) was administered based on multidisciplinary tumor board recommendations. Postoperative surveillance followed national guidelines and included clinical examination, imaging, and carcinoembryonic antigen (CEA) monitoring. The primary endpoints were DFS and OS. Secondary endpoints included oncological parameters such as the number of retrieved lymph nodes, circumferential resection margin (CRM), distal resection margin (R), perineural invasion, lymphovascular invasion, and short‐term outcomes like complications, conversion rate, and reoperation.

## 3 Statistical Analysis

Statistical analysis was conducted using IBM SPSS Statistics for Windows, Version 26.0 (IBM Corp., Armonk, NY, USA) [20]. Quantitative variables were assessed for normality using the Kolmogorov–Smirnov test. The sample size was not normally distributed. Continuous variables were expressed as mean ± standard deviation (SD). Categorical variables were reported as frequencies and percentages. Comparisons between groups were performed using the Chi‐square test, Fisher's exact test for categorical variables, and the Mann–Whitney U or Kruskal–Wallis tests for continuous variables, depending on data distribution. For survival analysis, the Kaplan–Meier method was applied to estimate OS and DFS, with log‐rank tests used to compare survival curves [21].

Univariate Cox proportional hazards regression was performed to identify potential prognostic factors. Significant variables in univariate analysis were entered into a multivariate Cox regression model to identify independent predictors of DFS and OS [22]. Hazard ratios (HRs) with corresponding 95% confidence intervals (CIs) and *p* values were calculated. A two‐sided *p* value < 0.05 was considered statistically significant.

## 4 Results

### 4.1 Baseline Characteristics

A total of 74 patients who underwent either laparoscopic (Group 1, *n* = 28) or robotic‐assisted (Group 2, *n* = 46) surgery for locally advanced rectal cancer were included. No statistically significant differences were observed between the two groups in terms of sex, tumor grade, tumor (T) and nodal (N) stage, perineural invasion (Pn), lymphatic invasion (L), venous invasion (V), type of resection, or resection margins (R). Patient demographics were comparable, with male predominance in both groups (Group 1: 60.7%, Group 2: 65.2%), as per (Table 1).

**TABLE 1:** Patient characteristics, pathological and surgical outcomes.

Non‐metastatic LARC (5 years) 2014–2018	Laparoscopic surgery G (1)		Robotic surgery G (2)		*p*
	Frequency	Percentage %	Frequency	Percentage %	
Sex					
Male	17	23.0	30	40.5	> 0.05
Female	11	14.9	16	21.6	
Grade					
G1	4	5.4	2	2.7	> 0.05
G2	23	31.1	41	55.4	
G3	1	1.4	3	4.1	
T					
T3	13	17.6	24	32.4	> 0.05
T4	15	20.3	22	29.7	
N					
N0	22	29.7	37	50.0	> 0.05
N1	3	4.1	8	10.8	
N2	3	4.1	1	1.4	
Resection margin (R)					
R0	27	36.5	45	60.8	> 0.05
R1	1	1.4	1	1.4	
Perineural Invasion (pn)					
Pn0	25	33.8	43	58.1	> 0.05
Pn1	3	4.1	3	4.1	
Lymph invasion (L)					
L0	19	25.7	33	44.6	> 0.05
L1	9	12.2	13	17.6	
Vascular Invasion (V)					
V0	25	33.8	41	55.4	> 0.05
V1	3	4.1	5	6.8	
Surgical technique					
Abdominoperineal resection (APR)	5	6.8	14	18.9%	> 0.05
End‐to‐end‐anastomosis (EEA)	15	20.3	18	24.3%	
EEA+ Covering colostomy	5	6.8	13	17.6%	
Low anterior resection (LAR) + colostomy	3	4.1	1	1.4%	
Neoadjuvant therapy	8	10.8	29	39.2%	**< 0.01**
Adjuvant therapy	16	21.6	33	44.6%	> 0.05
Conversion rate	1	1.4	4	5.4%	> 0.05
Relaparotomie	1	1.4	3	4.1%	> 0.05
(Continues)					

	Mean	Standard deviation	Mean	Standard deviation	*p*
Distance analverge	9.25	±4.12	7.49	±4.13	> 0.05
Number of harvested lymph nodes	16	±7	14	±5	**< 0.001**
Hospital stay	10.1	±5.0	8	±4.5	> 0.05

Bold values indicate statistical significance (*p* <0.05).

### 4.2 Surgical Parameters and Oncologic Quality

There were no statistically significant differences in mean hospital stay (Group 1: 10.1 ± 5.0 days vs. Group 2: 9 ± 4.5 days; *p* > 0.05) or conversion rates (3.6% vs. 8.7%, respectively; *p* > 0.05). The average tumor distance from the anal verge was not statistically significant, although slightly lower in the robotic group (7.5 ± 4.1 cm) compared to laparoscopic (9.3 ± 4.1 cm). A higher proportion of patients in the robotic group received neoadjuvant therapy (63% vs. 28.6%; *p* < 0.01). The number of retrieved lymph nodes was significantly lower in the robotic group (13.9 ± 4.9) compared to the laparoscopic group (16 ± 7; *p* < 0.001), although both values remained within acceptable oncologic standards, as shown in (Table 1).

### 4.3 Postoperative Complications

Postoperative complications, including urinary retention, showed no significant difference between the two groups. Surgical site infections, anastomotic insufficiency, and postoperative bleeding were comparable between the two groups (*p* > 0.05 in all). Notably, UTI rates were significantly lower in the robotic group (1.4% vs. 14.3%, *p* < 0.05). Rates of parastomal hernia, incisional hernia, intestinal obstruction, and stoma‐related complications did not differ significantly, as demonstrated in (Table 2). Relaparotomy was required in four patients overall (Group 1: 3.6%, Group 2: 8.7%; *p* > 0.05). Rates of endoscopic or vacuum‐assisted therapy were similar between groups. Re‐anastomosis of stomas was also not statistically significant, although performed more frequently in the robotic group (12.2% vs. 5.4%).

**TABLE 2:** Postoperative complications.

Postoperative complications	Laparoscopic group		Robotic group		*p*
	Frequency	Percentage %	Frequency	Percentage %	
Blood transfusion	5	6.8	3	4.1	> 0.05
Postoperative bleeding	3	4.1	2	2.7	> 0.05
Stoma complications	1	1.4	2	2.7	> 0.05
Parastomal hernia	0	0.0	2	2.7	> 0.05
Urinary complications	4	5.4	1	1.4	**< 0.05**
VAC therapie/Endoscopy	3	4.1	8	10.8	> 0.05
Surgical site infection	1	1.4	5	6.8	> 0.05
Anastomosis insufficiency	3	4.1	5	6.8	> 0.05
Recurrent tumor	0	0.0	2	2.7	> 0.05
Intestinal obstruction	2	2.7	4	5.4	> 0.05
Intra‐abdominal abscess	2	2.7	2	2.7	> 0.05

Bold values indicate statistical significance (*p* <0.05).

### 4.4 Oncologic Outcomes

R0 resection was similar (97.8%) in both groups. The lymph node involvement (N1/N2) and distant metastasis rates did not differ significantly. Tumor recurrence rates were low and comparable between groups. Notably, there was no difference in local recurrence (G1: 7.1%, G2: 10.9%) or distant metastases (G1: 25.0%, G2: 28.3%) (*p* > 0.05), as illustrated in (Table 3).

**TABLE 3:** Long‐term outcomes.

Long‐term outcomes	Laparoscopic group		Robotic group		*p*
	*n*	Percentage %	*n*	Percentage %	
Overall survival					
Alive	23	31.1	34	45.9	> 0.05
Died	5	6.8	12	16.2	
Lymph node recurrence (Regional recurrence)					
No	28	37.8	43	58.1	> 0.05
Yes	0	0.0	3	4.1	
Distant metastases					
No	21	28.4	33	44.6	> 0.05
Yes	7	9.5	13	17.6	
Local recurrence					
No	26	35.1	42	56.8	> 0.05
Yes	2	2.7	4	5.4	
Disease specific mortality					
No	22	29.7	33	44.6	> 0.05
Cardiopulmonary	1	1.4	6	8.1	
Cancer specific	5	6.8	6	8.1	
Others	0	0.0	1	1.4	

The univariate analysis shown in (Table 4) explores the relationship between clinical, pathological, and surgical parameters and patient mortality following surgery for locally advanced rectal cancer. The only variables significantly associated with increased mortality were *postoperative complications* (*p* < 0.001) and perineural invasion (Pn1) (*p* < 0.05), both recognized as strong indicators of poor prognosis in locally advanced rectal cancer. G3 tumors and recurrence showed a trend toward higher mortality, which did not reach statistical significance in this analysis, possibly due to the limited sample size. No other parameters—including sex, surgical technique, neoadjuvant therapy, or lymphovascular invasion—were significantly associated with mortality.

**TABLE 4:** Univariate analysis of clinical and surgical parameters associated with mortality in patients undergoing surgery for locally advanced rectal cancer (Comparison of demographic, oncologic, and perioperative variables between patients who survived and those who died during follow‐up. Statistical significance was assessed using Chi‐square or Fisher's exact test for categorical variables and independent t‐tests for continuous variables. *p*‐values < 0.05 were considered statistically significant).

Mortality specific parameters	Alive		Died		*p*
	Frequency	Percentage %	Frequency	Percentage %	
Study groups					
Gr. 1—Laparoscopic	23	31.1	5	6.8	> 0.05
Gr. 2—Robotic	34	45.9	12	16.2	
Grade					
G1	5	6.8	1	1.4	> 0.05
G2	51	68.9	13	17.6	
G3	1	1.4	3	4.1	
Complications					
No	23	31.1	0	0.0	**< 0.001**
Yes	34	45.9	17	23.0	
Sex					
Male	35	47.3	12	16.2	> 0.05
Female	22	29.7	5	6.8	
Pn. Invasion					
Pn0	53	71.6	15	20.3	**< 0.05**
Pn1	4	5.4	2	2.7	
Lymph invasion					
L0	41	55.4	11	14.9	> 0.05
L1	16	21.6	6	8.1	
Vascular invasion					
V0	50	67.6	16	21.6	> 0.05
V1	7	9.5	1	1.4	
Surgical procedure					
APR+ Colostomy	14	18.9	5	6.8	> 0.05
EE‐ Anastmose	27	36.5	6	8.1	
EEA+ c. colostomy	13	17.6	5	6.8	
LAR+ Colostomy	3	4.1	1	1.4	
Neoadjuvant therapy					
No	28	37.8	9	12.2	> 0.05
Yes	29	39.2	8	10.8	
Distance analverge	8.12	3.93	8.29	5.08	> 0.0
Conversion					
No	53	71.6	16	21.6	> 0.05
Yes	4	5.4	1	1.4	
Relaparotomy					
No	54	73.0	16	21.6	> 0.05
Yes	3	4.1	1	1.4	
Recurrent tumor					
No	56	75.7	16	21.6	> 0.05
Yes	1	1.4	1	1.4	

Bold values indicate statistical significance (*p* <0.05).

### 4.5 Survival Analysis

**FIGURE 1:**
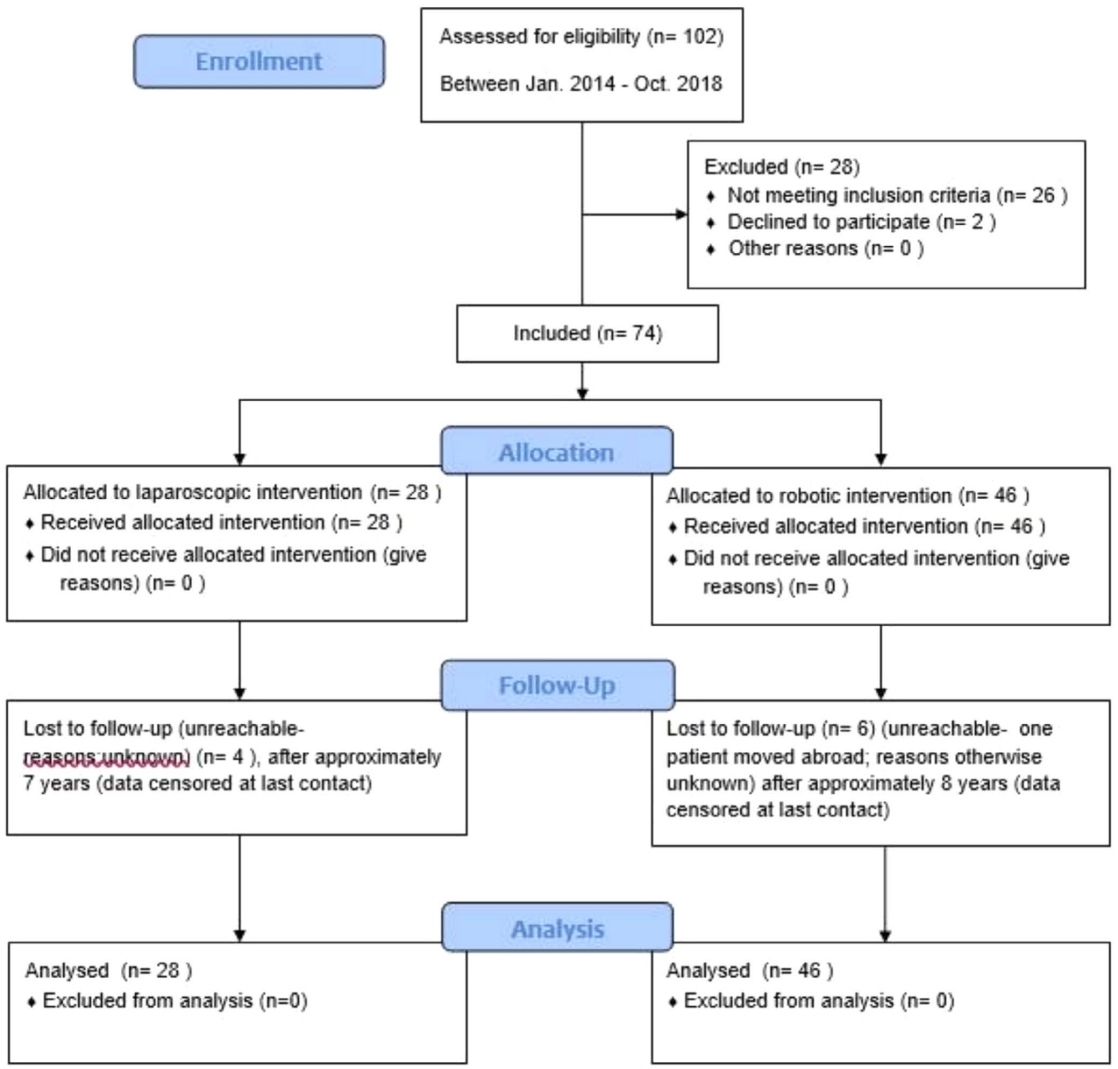
Flow diagram

The median follow‐up for the entire cohort was 125 months (range: 80–140 months). Ten patients (13.5%) were lost to follow‐up after approximately 7–8 years. These cases were censored in the survival analysis at the time of last known follow‐up to ensure accuracy in Kaplan–Meier estimates, as shown in the CONSORT Figure 1.

### 4.6 Overall Survival (OS)

**FIGURE 2:**
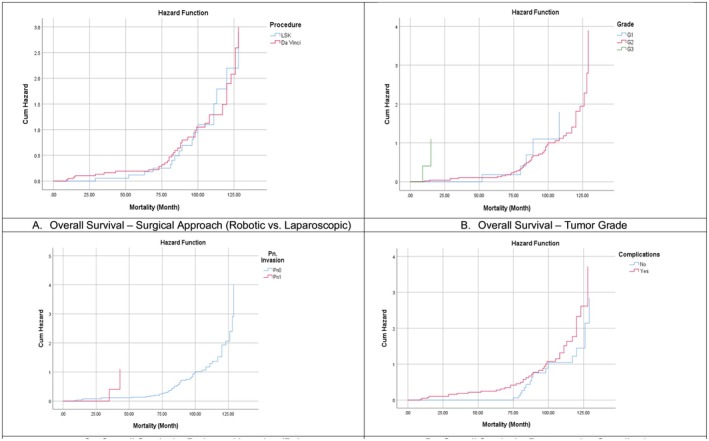
Overall survival Kaplan–Meier curves.

The multivariate Cox regression analysis revealed that robotic surgery was independently associated with significantly improved OS compared to laparoscopic surgery (AHR: 2.651, 95% CI: 1.171–6.004; *p* = 0.019). This supports the hypothesis that enhanced surgical precision and nerve‐sparing capabilities in robotic surgery may translate into better long‐term outcomes, as per Kaplan–Meier survival curve (Figure 2A).

High tumor grade (G3) and the presence of perineural invasion (Pn1) emerged as strong adverse prognostic indicators (AHR: 12.364 and 18.097, *p* < 0.05 for both), reinforcing their biological aggressiveness in rectal cancer, as shown in Kaplan–Meier survival curves (Figure 2B,C).

Notably, postoperative complications and comorbidities significantly increased mortality risk (AHR: 5.398; *p* < 0.001), emphasizing that complication management is a crucial determinant of long‐term survival, as shown in the Kaplan–Meier survival curve (Figure 2D).

Interestingly, patients undergoing restorative End‐to‐End anastomoses (EEA) had significantly better survival outcomes compared to those undergoing APR with end colostomy (*p* = 0.020 and *p* = 0.021, respectively), with hazard ratios below 0.3, suggesting benefits related to functional preservation or case selection. Other variables such as sex, conversion, relaparotomy, and tumor recurrence did not reach statistical significance in the adjusted model. However, they may still warrant attention in future analyzes with larger sample sizes, as shown in (Table 5).

**TABLE 5:** Multivariate cox regression analysis for overall survival in locally advanced rectal cancer.

Parameter	*p* (CHR)	CHR (95% CI)	*p* (AHR)	AHR (95% CI)
Study group (Robotic vs. Laparoscopic)	0.754	1.095 (0.620–1.935)	0.019	2.651 (1.171–6.004)
Tumor grade (G3 vs. G1)	0.001	14.273 (2.802–72.707)	**0.035**	12.364 (1.191–128.340)
Complications (Yes vs. No)	0.157	1.526 (0.849–2.742)	0.0	5.398 (2.171–13.420)
Sex (Female vs. Male)	0.765	1.088 (0.625–1.895)	0.668	1.174 (0.564–2.446)
Perineural invasion (Pn1 vs. Pn0)	0.011	5.054 (1.457–17.529)	**0.028**	18.097 (1.366–239.773)
Lymphatic invasion (L1 vs. L0)	0.023	2.125 (1.112–4.061)	0.757	1.182 (0.410–3.409)
Vascular invasion (V1 vs. V0)	0.77	1.191 (0.369–3.843)	0.078	0.066 (0.003–1.350)
Surgical procedure (EE‐Anastomosis vs. APR)	0.094	0.569 (0.295–1.101)	**0.026**	0.289 (0.097–0.862)
Surgical procedure (EEA + covering colostomy vs. APR)	0.762	1.125 (0.527–2.401)	**0.021**	0.297 (0.106–0.835)
Surgical procedure (LAR + Colostomy vs. APR)	0.121	3.359 (0.727–15.509)	0.218	3.822 (0.453–32.240)
Neoadjuvant therapy (Yes vs. No)	0.439	0.813 (0.481–1.374)	**0.018**	0.288 (0.103–0.804)
Conversion (Yes vs. No)	0.26	0.554 (0.198–1.547)	0.541	0.524 (0.066–4.164)
Relaparotomy (Yes vs. No)	0.265	0.512 (0.157–1.664)	0.399	0.423 (0.057–3.131)
Recurrent tumor (Yes vs. No)	0.192	3.874 (0.507–29.622)	0.096	14.449 (0.620–336.496)

*Note:* This table displays the comparative Cox proportional hazards regression results, showing the crude (CHR) and adjusted hazard ratios (AHR) with 95% confidence intervals (CI) and corresponding *p*‐values for key clinical and pathological variables. Significant predictors (*p* < 0.05) are highlighted to identify factors independently associated with overall survival. Bold values indicate statistical significance (*p* <0.05).

### 4.7 Disease‐Free Survival (DFS)

**FIGURE 3:**
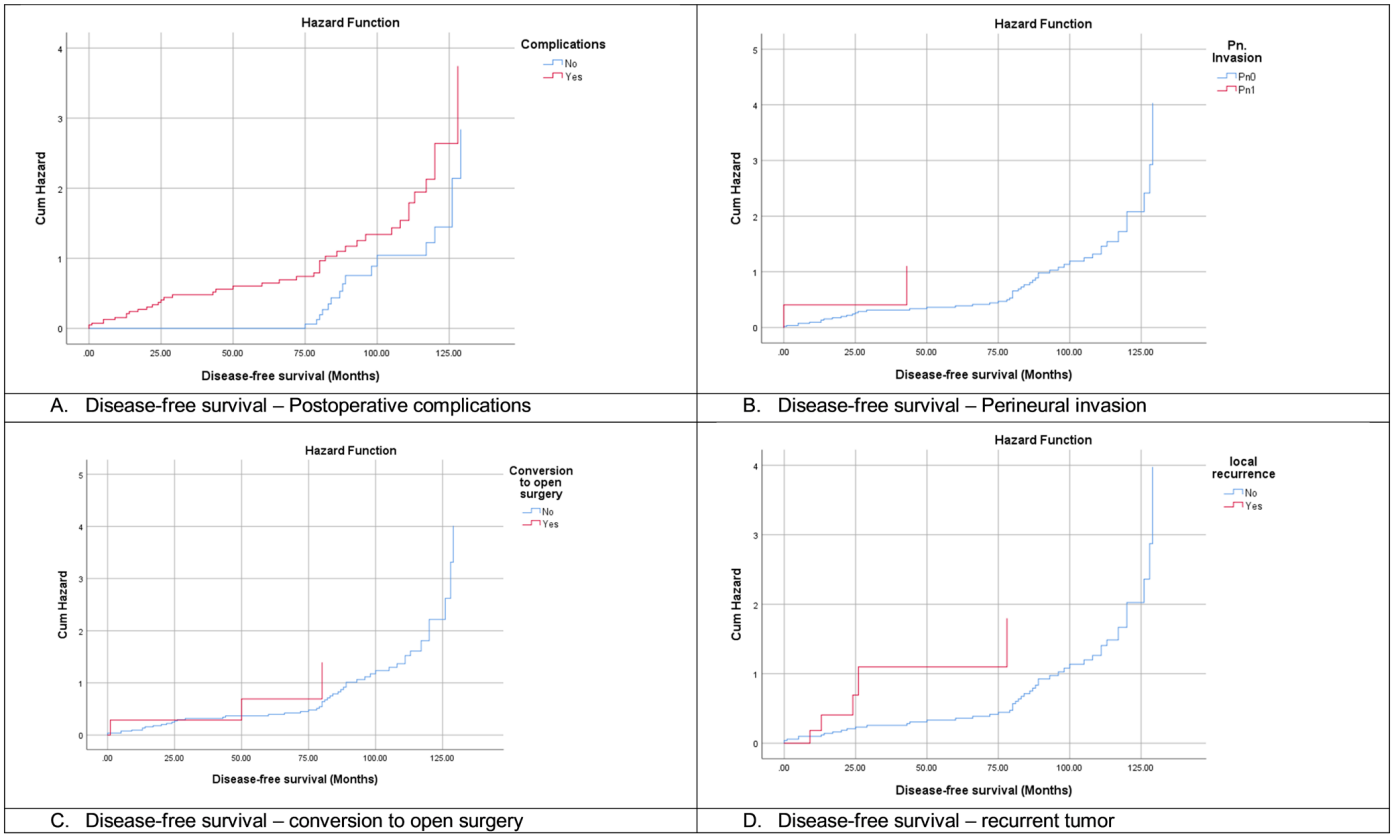
Disease‐free survival Kaplan–Meier curves.

In the multivariate Cox regression analysis for DFS, postoperative complications demonstrated a strong negative impact on DFS, with an adjusted hazard ratio (AHR) of 7.082 (95% CI: 2.922–17.166, *p* < 0.001), as per Kaplan–Meier survival curve (Figure 3A). This indicates that patients who experienced complications including anastomosis insufficiency, peritonitis, intra‐abdominal abscess, and postoperative admission to ICU with multiple organ failure (MOF) had a sevenfold increased risk of disease recurrence or death compared to those without complications, underscoring the critical importance of minimizing postoperative morbidity (Table 6).

**TABLE 6:** Multivariate cox regression analysis for disease‐free survival in locally advanced rectal cancer patients.

Variable	CHR	CHR 95% CI	*p*	AHR	AHR 95% CI	*p*
Robotic vs. Laparoscopic	1.041	0.595–1.820	0.889	1.635	0.698–3.827	0.258
Grade G2 vs. G1	0.699	0.294–1.664	0.418	0.713	0.251–2.022	0.524
Grade G3 vs. G1	**5.75**	1.178–21.852	**0.029**	6.477	0.746–56.251	0.090
Complications (Yes vs. No)	1.910	1.067–3.421	**0.030**	7.082	2.922–17.166	**0.000**
Female vs. Male	0.966	0.556–1.679	0.904	0.626	0.298–1.317	0.217
Pn1 vs. Pn0	3.031	0.917–10.020	0.069	**25.5**	1.994–324.911	**0.013**
L1 vs. L0	2.269	1.151–4.472	**0.018**	1.291	0.474–3.519	0.617
V1 vs. V0	1.860	0.564–6.138	0.308	0.031	0.002–0.564	0.019
EE‐anastomose vs. APR	0.551	0.285–1.066	0.077	**0.230**	0.077–0.688	**0.009**
EEA + Stoma vs. APR	1.103	0.521–2.335	0.797	**0.166**	0.055–0.500	**0.001**
TAR + Colostomy vs. APR	1.613	0.358–7.272	0.534	2.010	0.253–15.961	0.509
Neoadjuvant (Yes vs. No)	0.970	0.575–1.635	0.908	0.620	0.215–1.788	0.377
Distance to anal verge	1.016	0.954–1.082	0.618	1.072	0.956–1.203	0.234
Conversion (Yes vs. No)	0.884	0.313–2.498	0.815	**11.8**	1.385–99.919	**0.024**
Relaparotomy (Yes vs. No)	0.504	0.155–1.642	0.255	0.108	0.009–1.295	0.079
Recurrent tumor (Yes vs. No)	7.828	0.963–63.634	0.054	**200.4**	5.161–7783.843	**0.005**

*Note:* This table summarizes the results of multivariate Cox proportional hazards regression for disease‐free survival (DFS) in patients with locally advanced rectal cancer. It includes crude (CHR) and adjusted hazard ratios (AHR) with corresponding 95% confidence intervals (CI) and *p*‐values. Statistically significant predictors are highlighted.

Perineural invasion (Pn1 vs. Pn0) was also identified as a powerful adverse prognostic factor. Patients with Pn1 had a markedly higher risk of DFS events, with an AHR of 25.453 (95% CI: 1.994–324.911, *p* = 0.013), as per Kaplan–Meier survival curve (Figure 3B). Another notable finding was the association between conversion from minimally invasive to open surgery and worse DFS outcomes. Patients who required conversion had an AHR of 11.764 (95% CI: 1.385–99.919, *p* = 0.024), as shown in Kaplan–Meier survival curve (Figure 3C).

The presence of recurrent tumor was the most significant predictor of poor DFS in this analysis. The AHR was extremely high at 200.424 (95% CI: 5.161–7783.843, *p* = 0.005), as shown in the Kaplan–Meier survival curve (Figure 3D). Finally, surgical technique was the last key factor influencing DFS. Compared to abdominoperineal resection (APR), sphincter‐preserving approaches were associated with significantly improved outcomes. Specifically, end‐to‐end anastomosis (EEA) without a stoma had an AHR of 0.230 (95% CI: 0.077–0.688, *p* = 0.009), while EEA with a protective stoma had an even lower AHR of 0.166 (95% CI: 0.055–0.500, *p* = 0.001).

## 5 Discussion

Our study aimed to compare the long‐term oncologic outcomes of laparoscopic versus robotic‐assisted surgery in patients with locally advanced rectal cancer of the middle and lower third, and to evaluate independent prognostic factors affecting OS and DFS. Over a 10‐year follow‐up period, we retrospectively analyzed 74 patients, providing insight into the impact of surgical approach and postoperative variables on long‐term outcomes. The median follow‐up for the entire cohort was 125 months (range: 80–140 months). Notably, 91.9% of patients (*n* = 68) had ≥ 5‐year follow‐up, and 45.9% (*n* = 34) achieved ≥ 10‐year follow‐up.

Our analysis demonstrated no significant differences in baseline characteristics between the laparoscopic and robotic groups, suggesting appropriate group comparability. Tumor stage, nodal involvement, tumor grade, and resection margins were similar, which strengthens the internal validity of our outcome comparisons. These findings align with those reported by Kim et al. [23], who also found comparable baseline characteristics in a randomized clinical trial comparing robotic and laparoscopic rectal surgery.

Interestingly, the number of retrieved lymph nodes was significantly lower in the robotic group compared to the laparoscopic group, a difference that can be largely attributed to the significantly higher proportion of patients in the robotic group who received nCRT. It is well documented that nCRT can reduce lymph node yield due to treatment‐induced fibrosis, lymphoid depletion, and tissue scarring, making nodal dissection and identification more challenging [24, 25]. Despite this, both groups achieved oncologically adequate nodal retrieval, in line with established surgical standards [26].

Moreover, conversion rates were statistically comparable between the two groups. This is in line with the findings of Feng et al. [27], who also reported comparable conversion rates between robotic and laparoscopic surgery in rectal cancer patients. In contrast, Tian et al. [28] demonstrated a significantly lower conversion rate in the robotic group compared to the laparoscopic group, highlighting a potential advantage of robotic surgery in complex pelvic procedures. Notably, in our cohort, most conversions in the robotic group occurred during the first 2 years of the study period, suggesting a potential influence of the surgical learning curve of robotic platforms.

The hospital stay was not statistically significant between the groups. This trend has been noted in previous studies, such as those by Mayo et al. (2021), who demonstrated marginal reductions in postoperative stay with robotic surgery in rectal cancer patients [12, 29, 30]. However, institutional protocols, early mobilization programs, and complication rates may influence these findings [16, 31, 32, 33].

Regarding postoperative complications, differences were not statistically significant between the groups. Importantly, urinary complications, including incontinence and retention, were significantly lower in the robotic group, suggesting a potential benefit of robotic precision in pelvic dissection and nerve preservation. Similar findings have been reported in recent studies, which demonstrated that robotic techniques are associated with reduced rates of postoperative urinary dysfunction compared to laparoscopy in rectal cancer surgery [12, 34].

Although not statistically significant, relaparotomy and stoma reversal procedures were more frequent in the robotic group. These trends may reflect case selection bias, with more complex or advanced tumors managed robotically, and could also be influenced by the initial learning curve associated with the adoption of robotic surgery. The higher rate of stoma reversal may suggest a potential functional benefit of the robotic approach, although this finding contrasts with previous reports showing no significant difference between techniques [35, 36].

In terms of oncologic adequacy, both groups achieved high rates of R0 resection, and tumor recurrence rates, including local and distant recurrence, did not differ significantly. These findings support the oncological safety of both approaches and align with data from the ROLARR trial, which showed no difference in oncologic outcomes between laparoscopic and robotic TME [37].

Univariate analysis revealed that only postoperative complications (including anastomosis insufficiency, peritonitis, and renal failure), and perineural invasion (Pn1) were significantly associated with increased mortality. These findings reinforce their known prognostic significance in rectal cancer. Tumor grade G3 and recurrence were not statistically significant, possibly due to limited sample size. Similar associations have been documented by Martin Weiser et al. (2021) and Knijn et al. (2016), who identified histologic grade and Pn status as independent predictors of poor outcomes [38, 39]. This is further reinforced by our multivariate Cox regression analysis for OS, which confirmed tumor grade G3 and perineural invasion as strong adverse prognostic factors for OS.

Postoperative complications also emerged as a major contributor to mortality, underlining the need for complication prevention and optimized perioperative care—a conclusion consistent with findings from Glynne‐Jones et al. (2020) [40].

The multivariate Cox regression analysis for OS has demonstrated that robotic‐assisted surgery was independently associated with significantly improved OS compared to laparoscopic surgery, as visualized in Kaplan–Meier analysis. This finding may reflect enhanced 3D visualization, improved ergonomics, and greater precision during dissection, particularly in the deep pelvis. A similar survival advantage in robotic surgery has been reported in a recent meta‐analysis of 19,731 patients by Safiejko et al. [41]. In contrast, Kim et al. [42] found comparable long‐term outcomes between robotic and laparoscopic approaches.

Interestingly, patients undergoing restorative EEA with or without protective stoma had significantly improved OS compared to those undergoing APR. Hazard ratios were low, suggesting a substantial survival benefit. However, this finding may also reflect selection bias, as patients selected for restorative procedures often present with smaller or more favorably located tumors at baseline. The observed benefit may also be attributable to better functional outcomes and more favorable tumor biology. These results are consistent with the findings of Denost et al. [43], who demonstrated that sphincter‐preserving procedures, when oncologically safe, improve long‐term outcomes. Type of surgery remains an important factor, but its interpretation should consider selection bias—for example, patients undergoing APR must have presented with more locally advanced or technically challenging tumors.

Regarding DFS, postoperative complications remained a strong predictor of recurrence or death. This highlights the critical role of postoperative recovery in long‐term cancer control. Complications such as anastomotic leakage, peritonitis, and MOF significantly impact DFS, a conclusion echoed by Watanabe et al. [44], who emphasized the link between early postoperative complications and tumor recurrence.

Perineural invasion was again confirmed as a robust independent risk factor for poor DFS, further validating its clinical importance. Conversion from minimally invasive to open surgery also significantly worsened DFS, indicating that conversion may be a surrogate marker for complex cases or suboptimal surgical exposure, aligning with findings from Allaix et al. (2020) and Abdalla et al. (2022) [45, 46].

Most notably, tumor recurrence was the most significant predictor of poor DFS in this analysis, reaffirming the aggressive nature of recurrence and its devastating impact on patient outcomes [47, 48, 49].

Surgical technique continued to influence DFS: patients undergoing restorative procedures had significantly better outcomes than those undergoing APR. Specifically, EEA without stoma and with protective stoma both conferred significant DFS benefits, consistent with functional preservation theories and possibly indicating favorable selection bias. These results are in agreement with Denost et al.'s findings and Stevenson et al. [50, 51].

Other parameters such as sex, conversion, relaparotomy, and recurrence did not reach statistical significance in the multivariate analysis. However, the observed trends—particularly with relaparotomy and conversion—suggest potential associations that merit further investigation in larger, multicentre prospective trials.

### 5.1 Limitations

This study is subject to several limitations. First, its retrospective design introduces the risk of selection bias and limits causal inference. Second, the relatively small sample size (*n* = 74), particularly after propensity score matching, may reduce statistical power and limit the generalizability of certain subgroup findings (e.g., recurrence, conversion, stoma reversal). Third, loss to follow‐up in some patients may have affected the accuracy of long‐term survival analysis. Additionally, although all procedures were performed by a single experienced surgical team following standardized protocols, unmeasured confounders such as surgeon experience curve, robotic platform learning curve, or patient compliance may still influence outcomes.

The use of the Da Vinci Si system and the lack of total neoadjuvant therapy (TNT) in our cohort reflect the standard of care during the study period. Although newer technologies and oncologic paradigms have since emerged, our findings remain clinically relevant and representative of real‐world outcomes in early robotic adoption. The exclusion of more recent cases was intentional to minimize heterogeneity due to learning curves, platform transitions, and surgeon variability.

Lastly, the inclusion of various resection types (e.g., APR, LAR, EEA) adds procedural heterogeneity, which may influence outcomes and complicate direct comparisons.

## 6 Conclusion

In this 10‐year retrospective analysis, both laparoscopic and robotic‐assisted approaches were found to be oncologically safe and effective for the treatment of locally advanced middle and low rectal cancer. However, robotic‐assisted surgery was associated with a statistically significant improvement in OS and a lower incidence of urinary complications, suggesting potential benefits in terms of both oncologic and functional outcomes. Independent predictors of poor prognosis included high tumor grade, perineural invasion, postoperative complications, conversion to open surgery, and tumor recurrence. These findings highlight the importance of meticulous surgical technique, complication prevention, and careful patient selection. Restorative procedures, when oncologically feasible, were associated with better long‐term outcomes compared to APR. While robotic surgery appears to offer specific advantages, it is important to note that comparable outcomes can be achieved with laparoscopic surgery when performed by experienced surgeons. Further prospective, randomized studies with larger cohorts are warranted to confirm these results and define the optimal role of robotic surgery in the management of rectal cancer.

## Author Contributions

**Ahmed Abdelsamad:** conceptualization (lead), data curation (lead), formal analysis (lead), investigation (lead), methodology (lead), project administration (lead), resources (equal), supervision (lead), validation (lead), visualization (lead), writing – original draft (lead), writing – review and editing (lead). **Seyidali Mirzazada:** data curation (equal), investigation (equal), methodology (equal), software (equal). **Karsten Ridwelski:** investigation (equal), resources (equal), supervision (equal), validation (equal), visualization (equal), writing – original draft (equal), writing – review and editing (equal). **Mohamad Nour Nasif:** formal analysis (equal), investigation (equal), methodology (equal), resources (equal), software (equal), visualization (equal). **Florian Gebauer:** investigation (equal), methodology (equal), project administration (equal), resources (equal), supervision (equal), validation (equal), visualization (equal), writing – review and editing (equal).

## Funding

The authors have nothing to report.

## Ethics Statement

This retrospective study was conducted following the Declaration of Helsinki. Ethical approval was waived by our Institutional Review Board (IRB), as the study involved only anonymized patient data collected during routine clinical care. The requirement for written informed consent was also waived.

## Conflicts of Interest

The authors declare no conflicts of interest.

## Data Availability

The data that support the findings of this study are available on request from the corresponding author. The data are not publicly available due to privacy or ethical restrictions.
